# Supplementary material for: MMP2 Modulates Inflammatory Response during Axonal Regeneration in the Murine Visual System
Source: Cells. 2021 Jul 2;10(7):1672. doi: 10.3390/cells10071672 (PMC8307586; doi:10.3390/cells10071672)
Supplement: Supplementary file 1 [file cells-10-01672-s001.zip › cells-1245582-supplementary.pdf]

Table S1: Repopulation of myeloid cells in the peripheral blood of transplanted mice. Percentage of CD45.1$^+$ myeloid cells and CD45.2$^+$ myeloid cells on the total amount myeloid cells in the peripheral blood shows a repopulation efficiency of over 99%. BM: bone marrow, WT: wild-type.

**Condition**	**CD45.1$^+$ myeloid cells (%)**	**CD45.2$^+$ myeloid cells (%)**
**WT + WT BM**	99,64±0,09	0,36±0,09
***Mmp2$^{-/-}$ + Mmp2$^{-/-}$* BM**	0,008±0,004	99,99±0,004
**WT *+ Mmp2$^{-/-}$* BM**	99,77±0,11	0.23±0,11
***Mmp2$^{-/-}$* + WT BM**	0,45±0,13	99,56±0,13

Table S2: Primer sequences using SYBR green assays and the optimized annealing temperatures for qRT-PCR of the different genes.

Gene	Forward primer	Reverse primer	Annealing temperature (°C)
**Ywhaz**	CTGACCTACAATGCCTCCATC	CATCCTGCTGCCATTGCTTA	60
**Top1**	CGTACCTACAATGCCTCCATC	AGAATTGCAACAGCTCGATTG	60
**TNF**	ACCCTGGTATGAGCCCATATAC	ACACCCATTCCCTTCACAGAG	56
**IL1b**	CTCTTGTTGATGTGCTGCTG	GACCTGTTCTTTGAAGTTGACG	54
**IFNg**	CCAGGCAGCGTGTTCCT	GTACCTACAATGCCTCCATC	58
**IL10**	AGGAACTCAAGCGGGATATG	GAGGCTAGATACTGCTCGAT	60
**Ym1**	CAAGTCAAATTCAGAGCATACTTCG	TGAGCTTCAGACTTCGGTGAA	60
**iNOS**	GCGACTGGTGGGAGTTTTG	AGCACCTCTTCTTGTCCGTTG	60
**IL6**	GCTACACTGGCTACACTCTT	TCGCCAAGGAGACTCTTTAC	60
**CNTF**	TGGCTAGCAAGGAAGATTCGTT	CCCATAATGGCTCTCATGTGC	60
**MMP2**	AACTACGATGATGACCGGAAGTG	TGGCATGGCCGAACTCA	60
**GAP43**	CCACTGATAACTCCCCGTCC	CTCGGGGTCTTCTTTACCCT	60
